# Protective Effects of PollenAid Plus Soft Gel Capsules’ Hydroalcoholic Extract in Isolated Prostates and Ovaries Exposed to Lipopolysaccharide

**DOI:** 10.3390/molecules27196279

**Published:** 2022-09-23

**Authors:** Annalisa Chiavaroli, Simonetta Cristina Di Simone, Alessandra Acquaviva, Maria Loreta Libero, Claudia Campana, Lucia Recinella, Sheila Leone, Luigi Brunetti, Giustino Orlando, Nilofar, Irene Vitale, Stefania Cesa, Gokhan Zengin, Luigi Menghini, Claudio Ferrante

**Affiliations:** 1 Department of Pharmacy, Botanic Garden “Giardino dei Semplici”, “G. d’Annunzio” University, via dei Vestini 31, 66100 Chieti, Italy; 2 Department of Drug Chemistry and Technology, Sapienza University of Rome, 00185 Rome, Italy; 3 Department of Biology, Science Faculty, Selcuk University, Konya 42130, Turkey

**Keywords:** Graminex pollen, hydroalcoholic extract, phenolic compounds, inflammation, oxidative stress, gene expression, TRPV1

## Abstract

Pollen extract represents an innovative approach for the management of the clinical symptoms related to prostatitis and pelvic inflammatory disease (PID). In this context, the aims of the present work were to analyze the phenolic composition of a hydroalcoholic extract of PollenAid Plus soft gel capsules, and to evaluate the extract’s cytotoxic effects, in human prostate cancer PC3 cells and human ovary cancer OVCAR-3 cells. Additionally, protective effects were investigated in isolated prostate and ovary specimens exposed to lipopolysaccharide (LPS). The phytochemical investigation identified catechin, chlorogenic acid, gentisic acid, and 3-hydroxytyrosol as the prominent phenolics. The extract did not exert a relevant cytotoxic effect on PC3 and OVCAR-3 cells. However, the extract showed a dose-dependent inhibition of pro-inflammatory IL-6 and TNF-α gene expression in prostate and ovary specimens, and the extract was effective in preventing the LPS-induced upregulation of CAT and SOD gene expression, which are deeply involved in tissue antioxidant defense systems. Finally, a docking approach suggested the capability of catechin and chlorogenic acid to interact with the TRPV1 receptor, playing a master role in prostate inflammation. Overall, the present findings demonstrated anti-inflammatory and antioxidant effects of this formulation; thus, suggesting its capability in the management of the clinical symptoms related to prostatitis and PID.

## 1. Introduction

Prostatitis and pelvic inflammatory disease (PID) are common chronic conditions in the population, caused by pathogenic infection [1,2]. Herbal extracts endowed with antioxidant/anti-inflammatory effects have been long considered as a reliable strategy to blunt the burden of oxidative stress and inflammation in prostate and ovary tissue [3,4,5]. Pollen extract represents an innovative approach for the management of the clinical symptoms related to prostatitis [6], being also able to relieve inflammation and hyperplasia of the prostate [7], with anticancer potential most likely associated with antioxidant and antimutagenic effects [8]. In this case, pollen appears to relieve pain in patients with benign prostatic hyperplasia, at least in the early stages. Its administration together with chemotherapeutic agents has been seen to increase the number of people who have experienced a significant therapeutic effect [9]. Due to its content in phytoestrogens, pollen has also been shown to improve the symptoms of polycystic ovary syndrome in rats [10], although there is still a lack of scientific literature about the effects of pollen in PID.

Pollen represents the set of microgametophytes produced by spermatophytes in the male cones, in the case of gymnosperms, and in the anthers, the fertile part of the stamens, in the case of angiosperms. Pollen has different shapes and colors and dimensions between 2.5 and 250 µm. Its composition varies mainly according to the geographical origins and the botanical species visited by the insect, together with less relevant but still important factors such as climatic conditions and the type of soil [8]. From the literature, it appears that pollen grains deriving from various plant species contain about 200 active substances, including proteins and amino acids, carbohydrates, lipids and fatty acids, enzymes and coenzymes, nucleic acids, phenolic compounds, vitamins, and minerals [9].

Pollen, as well as other bee products, has been used since ancient times as a food for its nutritional value and for a wide spectrum of therapeutic activities, of which the best known are antifungal, antibacterial, antiviral, antioxidant, and anti-inflammatory. Evidence has been reported on the activity of phenolic compounds in pollen extracts against Gram-positive and Gram-negative bacteria, fungi, and yeasts [10,11,12]. The content in phenolic compounds has been also related to the anti-inflammatory and antioxidant properties of pollen [13].

In this context, the aims of the present work were to analyze the phenolic composition of an innovative formulation containing Graminex G60^TM^ Flower Pollen Extract, a mixture of standardized and dry pollen of rye grass (*Secale cereale* L.), corn (*Zea mays* L.), and timothy (*Phleum pratense* L.), and NAX^TM^ 7% paste, both suspended in extra virgin olive oil (EVO) as amber soft gel capsules (PollenAid Plus), and to evaluate cytotoxic activity of the hydroalcoholic extract from this formulation on immortalized human prostate cancer PC3 cells and human ovary cancer OVCAR-3 cells. The effect of the extract on cell viability was also investigated in a myoblast C2C12 cell line, which was chosen as a non-tumor comparison cell model. The protective effects of this extract were also investigated in isolated prostate and ovary tissues exposed to *Escherichia coli* lipopolysaccharide (LPS), a reliable experimental model of tissue inflammation [14]. In this context, we measured the gene expression of pro-inflammatory factors, including inteleukin-6 (IL-6) and tumor necrosis factor α (TNF-α). The gene expression of superoxide dismutase (SOD) and catalase, which are deeply involved in antioxidant response, was measured in both tissues, as well. Finally, an in silico study was conducted for unraveling, albeit partially, the mechanisms of action underlying the observed effects and putative interactions against transient receptor potential vanilloid 1 (TRPV1), an ion channel present on sensory neurons and localized in particular on small neurons and type C amielin fibers responsible for nociceptive transmission. TRPV1 is activated by a number of harmful stimuli and its activity is regulated by numerous inflammatory mediators including prostaglandins, bradykinins, and serotonin. In addition to the sensitization of TRPV1 by inflammatory mediators, the activation of TRPV1 stimulates the release of inflammatory molecules associated with the transmission of pain such as substance P and bradykinin, which in turn contribute to the peripheral sensitization of TRPV1 as well as the activation of mast cells and the perpetuation of the state of neurogenic inflammation. The administration of substances capable of acting on some of the actors underlying the pathophysiology of pain, such as TRPV1, and at the same time counteracting neurogenic inflammation is a rational approach in the treatment of chronic pelvic pain pathologies.

## 2. Results

**Figure 1:**
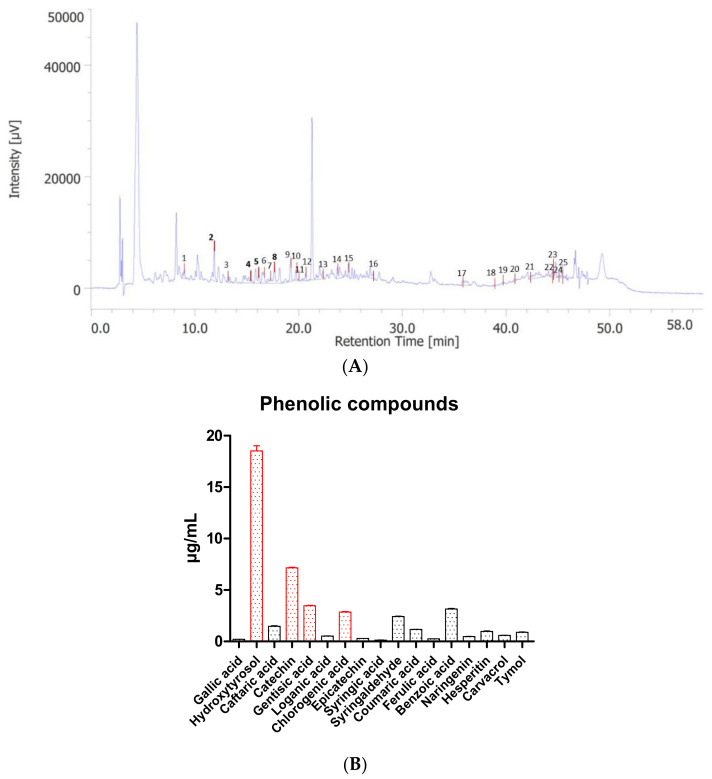
(**A**) Chromatogram related to the analysis of the hydroalcoholic extract from Graminex pollen. (**B**) Phenolic compounds identified and quantified in the extract. 3-Hydroxytyrosol (peak #2), catechin (peak #4), gentisic acid (peak #5), and chlorogenic acid (peak #8) were the most abundant phenolic compounds present in the extract.

In the present study, 17 compounds were identified in the hydroalcoholic extract and quantified through HPLC-DAD-MS. The quantification was carried out by comparison with pure standards (Figure 1). Among assayed compounds, 3-hydroxytyrosol, catechin, gentisic acid, and chlorogenic acid were the main phytochemicals (Table S1). The quantification of such compounds in the extract is consistent with their previous identification in the plants of origin of the pollen [15,16], despite the presence of the vehicle (extra-virgin olive oil: EVO); thus, indicating the EVO as a reliable vehicle which displays multiple advantages: biocompatibility, health-promoting effects, and sustainability. The determination of phenolic compounds is consistent with our previous study of Graminex pollen using different analytical conditions [17]. The presence of phenolic compounds in the extract makes rational the evaluation of protective effects in prostate and ovary cells and tissues, as described below.

**Figure 2:**
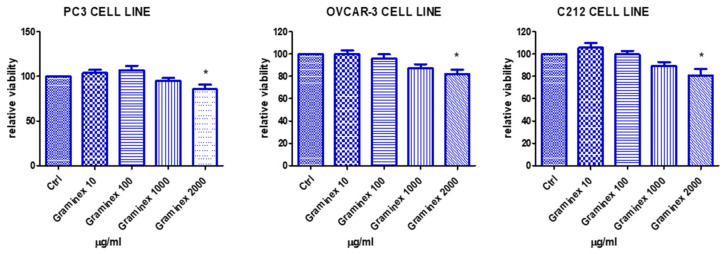
Effects of the extract on human prostate cancer PC3 cells, human ovary cancer OVCAR-3 cells, and the non-tumoral C2C12 myoblast cell line. At the highest tested concentration, the extract induced a mild reduction of cell viability in all tested cell lines (ANOVA, *p* < 0.05; * *p* < 0.05 vs. ctrl group). However, the cell viability was always over 70% compared to the respective ctrl group; thus, suggesting biocompatibility in the concentration range 10–2000 µg/mL. This range was considered as biocompatible for the subsequent ex vivo determination in the prostate and ovary tissues.

Regarding the pharmacological study, the extract (10–2000 µg/mL) was tested on different cell lines, namely human prostate cancer PC3 cells and human ovary cancer OVCAR-3 cells, to investigate cytotoxic properties against tumor cells. Additionally, the extract was also added to the medium of myoblast C2C12 cells, to determine the susceptibility of a non-tumor cell line to scalar concentrations. Intriguingly, all three cell lines displayed a similar response after exposure to the extract. Indeed, the cell viability was slightly reduced at the highest tested concentration (2000 µg/mL). However, the cell viability was >70% compared to the control (ctrl) group, in all three cell models (Figure 2); thus, ruling out any significant cytotoxic effect towards both tumor and non-tumor cells.

**Figure 3:**
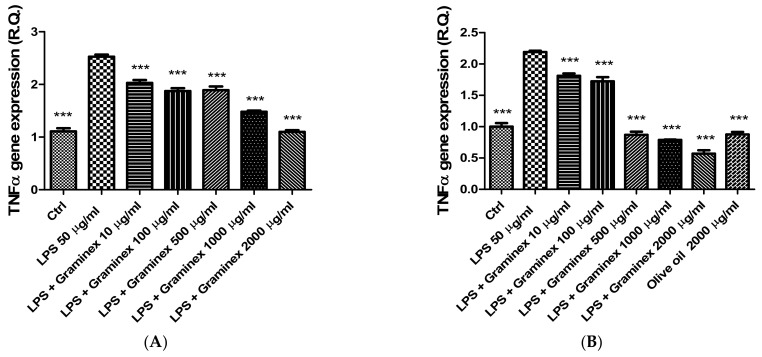
Inhibitory effects of the hydroalcoholic extract (10–2000 µg/mL) on TNF-α gene expression in isolated prostate (**A**) and ovary (**B**) specimens. ANOVA, *p* < 0.0001; *** *p* < 0.001 vs. ctrl group.

**Figure 4:**
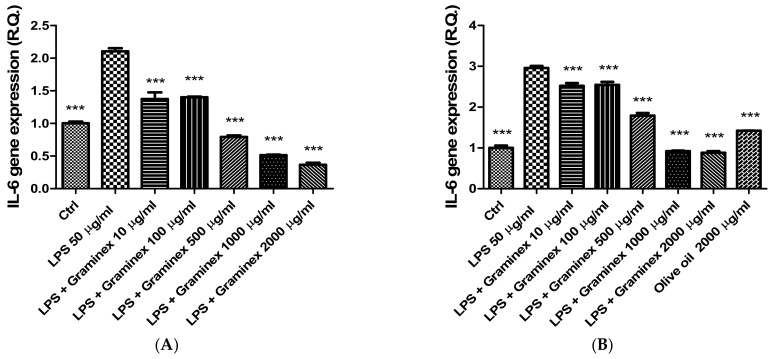
Inhibitory effects of the hydroalcoholic extract (10–2000 µg/mL) on IL-6 gene expression in isolated prostate (**A**) and ovary (**B**) specimens. ANOVA, *p* < 0.0001; *** *p* < 0.001 vs. ctrl group.

The extract (10–2000 µg/mL) was also tested in isolated prostate and ovary specimens challenged with *E. coli* LPS, chosen as pro-inflammatory stimulus [14,17]. In this context, it is notable that *E. coli* infection has been related to both prostatitis and PID [18,19]. The LPS stimulus induced the upregulation of TNF-α and IL-6 both in prostate and ovary tissues (Figure 3 and Figure 4). The extract treatment was effective in reverting the increased gene expression of both cytokines; thus, demonstrating anti-inflammatory effects in both tissues. In the case of prostate tissues, this study is also consistent with previous clinical observations about the capability of Graminex pollen to contrast the inflammatory component of prostatitis [20,21]. The anti-inflammatory effects also agree with previous studies highlighting the inhibition of IL-8 production [22] and cyclooxygenase (COX)-2 and inducible nitric oxide synthase (iNOS) activities, measured as prostaglandin E_2_ and nitrites levels, respectively [23,24,25].

**Figure 5:**
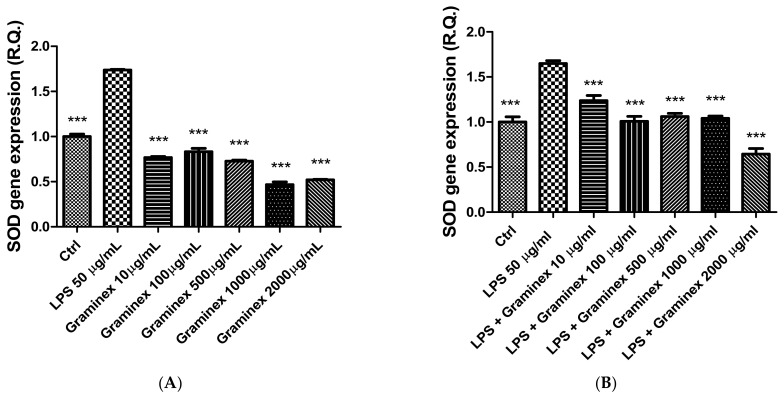
Inhibitory effects of the hydroalcoholic extract (10–2000 µg/mL) on SOD gene expression in isolated prostate (**A**) and ovary (**B**) specimens. ANOVA, *p* < 0.0001; *** *p* < 0.001 vs. ctrl group.

**Figure 6:**
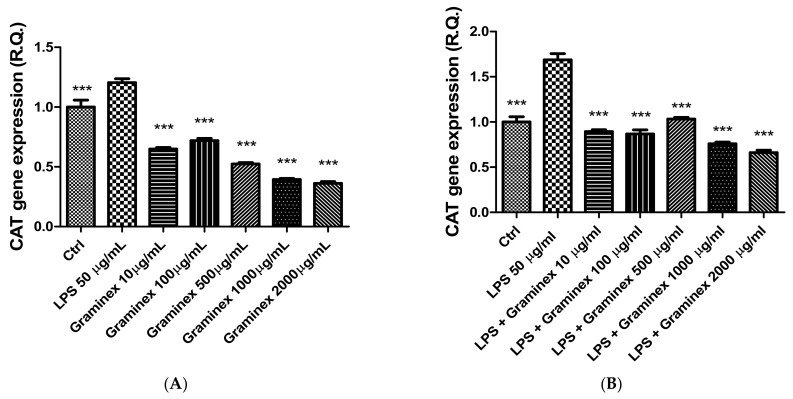
Inhibitory effects of the hydroalcoholic extract (10–2000 µg/mL) on CAT gene expression in isolated prostate (**A**) and ovary (**B**) specimens. ANOVA, *p* < 0.0001; *** *p* < 0.001 vs. ctrl group.

In prostate and ovary specimens, LPS stimulus (50 µg/mL) was also effective in increasing the gene expression of both CAT and SOD (Figure 5 and Figure 6), which are deeply involved in the antioxidant response. Indeed, the extract was able to prevent the LPS-induced upregulation of CAT and SOD gene expression. Additionally, after extract administration, the gene expression of both enzymes was even lower than the one displayed by the control (ctrl) group. Previously, LPS stimulus has been found to alter CAT and SOD levels, with both inhibitory and stimulatory effects. We cannot exclude that these discrepancies could depend, albeit partially, on the employed experimental models [26,27]. Therefore, also considering the intrinsic scavenging/reducing properties and ability of Graminex to blunt LPS-induced lipoperoxidation in isolated prostate [17], we hypothesize that the extract effects on SOD and CAT gene expression could be related to antioxidant effects, which can be mediated, albeit partially, by polyphenolic compounds.

**Figure 7:**
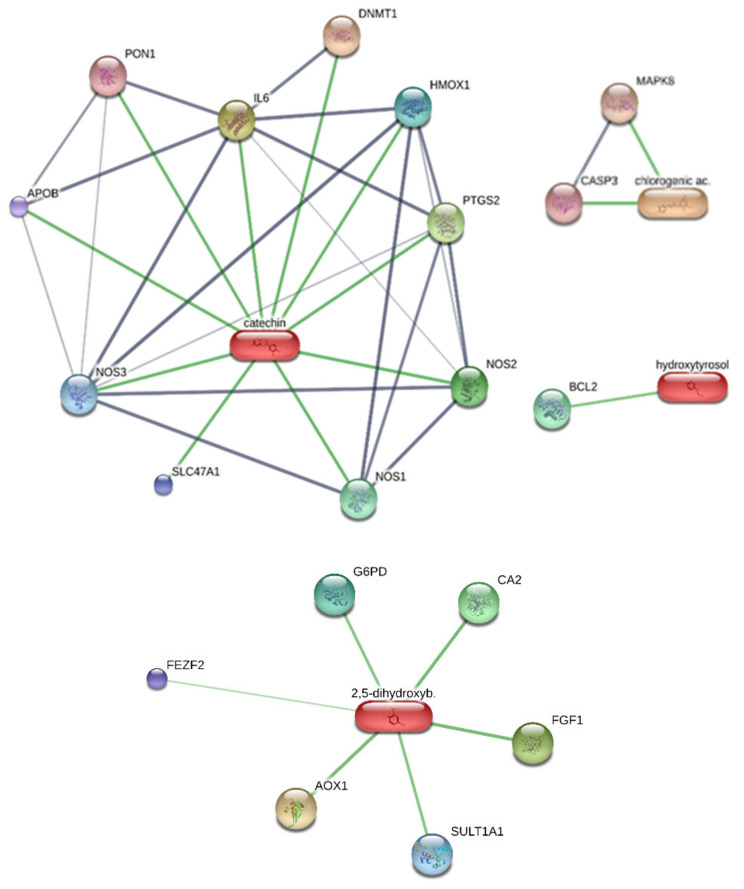
Target component analysis conducted on the STITCH bioinformatics platform for unraveling putative interactions between prominent extracts’ phytochemicals and putative proteins involved in inflammatory and cytotoxicity effects.

In order to explore the mechanisms of action underlying the observed effects, an in silico study was conducted on the platform STITCH, considering the main phytochemicals present in the extract; namely catechin, 3-hydroxytyrosol, chlorogenic acid, and gentisic acid (2,5-dihydroxybenzoic acid). Catechin was predicted to interact with IL-6, cyclooxygenase-2 (COX-2, PGTS2), and with iNOS (Figure 7). This is partly consistent with our findings of anti-inflammatory effects by the extract, in both prostate and ovary tissue, and with the literature data [5]. Intriguingly, 3-hydroxytyrosol and chlorogenic acid were predicted to interact with BCL-2 and caspase-3, respectively. Previous studies showed the capability of 3-hydroxytyrosol and chlorogenic acid to reduce BCL-2 and caspase-3 gene and protein expression, respectively [28,29], while gentisic acid could interact with fibroblast growth factor 1 (FGF1), whose levels are increased in prostate cancer [30]. This could explain, albeit partially, the mild reduction (<30%) of cell viability in all considered cell lines at the highest tested concentration.

**Figure 8:**
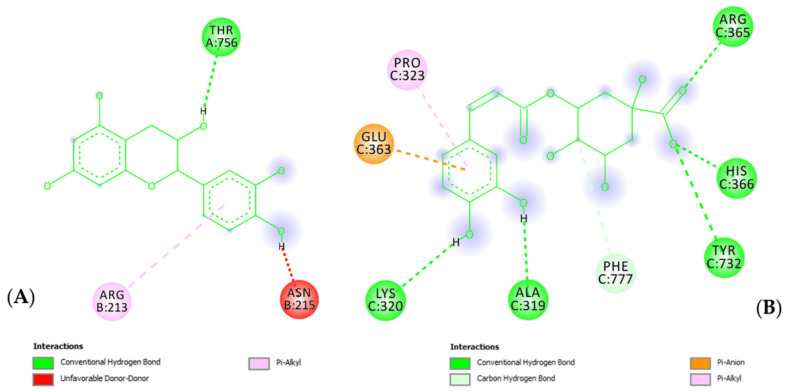
(**A**) Putative interactions between catechin and TRPV1 receptor (PDB ID: 7LR0). Free energy binding (∆G) and putative affinity (*K*_i_) are −6.8 kcal/mol and 10.5 µM, respectively. (**B**) Putative interactions between chlorogenic acid and TRPV1 receptor (PDB ID: 7LR0). Free energy binding (∆G) and putative affinity (*K*_i_) are −6.7 kcal/mol and 12.5 µM, respectively.

Finally, a docking approach was conducted to explore the putative interactions between the extract’s phytochemicals and the TRPV1 receptor, whose expression is increased in prostate inflammation [31]. Among phytochemicals detected in the extract, chlorogenic acid and catechin showed micromolar affinity (10–12 µM) towards the TRPV1 receptor (Figure 8); thus, suggesting direct interactions that could be crucial in mediating the observed anti-inflammatory properties. According to these predictions, further in vitro studies are needed to unravel the effects of catechin and chlorogenic acid on TRPV1 expression and activity.

### Color Analysis

The data obtained by the color analysis of the olive oil used as a vehicle and of the formulated product used to fill the soft capsules are reported in Table 1. The high luminance (70.46) of the pale yellow extra-virgin olive oil was changed to a very low value (14.96), which accounts for the very dark brown color of the formulated product. This drastic change, not shown by the only a* parameter, a weak red parameter, was accompanied by a drastic decrease of b* (positive, yellow parameter) and correlated saturation (C*ab), as well as the nuance turning from pale yellow to dark orange.

The calculated color differences of the Graminex G60^TM^ Flower Pollen Extract used for the formulation showed a much lower luminance (ΔL*, −71), a little redder color and a more yellow sample, much darker and browner, but less opaque in respect to the pollen powder used in the formulation, whose CIEL*a*b* parameters, reported in our previous work [15], were L* 86.05; a* 1.71; b* 11.96; C*_ab_ 12.09; h_ab_ 81.88. On the other hand, if slightly less important differences were shown between the formulated product and the extra virgin olive oil in terms of L* (ΔL* −55), and not-relevant changes of a* were registered, more significant differences were, in contrast, shown by the b* parameter (Δb*, −98) so that, on the whole, the sample appeared opaque, dark, and completely without color. 

It seems of particular concern to compare matrices so different in superficial characteristics (solid powder, oily, sticky paste) and coming from different compositions and mixtures. Moreover, to our knowledge only one study is available, in which microscopic analysis, NIR spectroscopy, e-nose and e-tongue methods, as well as color analysis, were applied to perform a discrimination of bee pollens. As the authors reported, chemical composition largely depended on the botanical origin and can change due to the oxidation process. Authors also reported that dominance of positive a* and b* parameters could account for carotenoid and flavonoid compounds [32].

**Figure 9:**
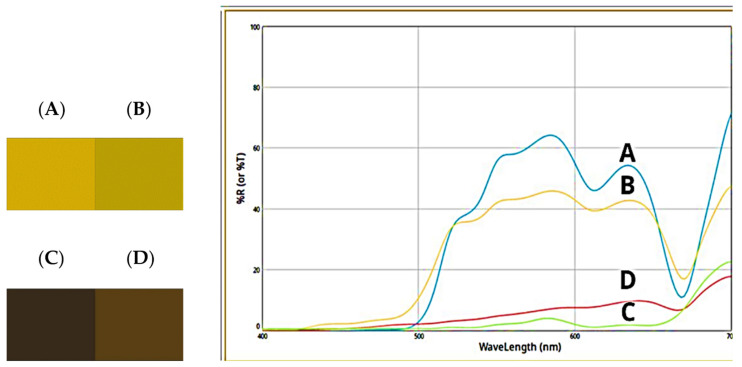
Color palette of olive oil ((**A**): t°; (**B**) t 9 months) and of the formulated product ((**C**): t°; (**D**): t 9 months) and relative reflectance curves.

This statement agrees with the analyses we performed on the carrier oil and on the formulation after 9 months of storage (Figure 9), which showed slight modification of oil, towards a greener color, and of the formulation to a less intense brown, which could both account for a slight discoloration of carotenoids, both coming from the oil and from the pollen in the case of the formulated mixture. We reported in our previous works the carotenoid bleaching in powder infant formulas and in powder allium samples evaluated by color analyses [33,34].

## 3. Material and Methods

### 3.1. Samples

PollenAid Plus soft gel capsules were kindly provided by IdiPharma (Catania, Italy). The formulation contains Graminex G60™ Flower Pollen Extract (45.86%), NAX™ 7% paste (2.29%), soy lecithin (3.06%), yellow beeswax (2.93%), and extra virgin olive oil (45.86) as the vehicle. All the ingredients are contained in oval dark amber capsules. Graminex G60^TM^ Flower Pollen Extract is a water-soluble extract of rye pollen grown in the USA. It is standardized to 6% amino acids and exhibits both antioxidant and anti-inflammatory activities. It is also non-allergenic, solvent-free, vegan, and non-GMO. Graminex G60™ Flower Pollen Extract was studied in our previous paper [17]. NAX™ 7% paste is a lipid-soluble paste of rye pollen grown in the USA. It is standardized to 7% phytosterols including β-sitosterol plus essential fatty acids such as ω-3 and ω-6. Applications include women’s health, skin health, and heart health, and it can be directly formulated into soft gels. It is non-allergenic, solvent-free, vegan, and non-GMO. The extraction of this formulation was carried out by diluting 100 µL in 1 mL of a hydroalcoholic solution, constituted by 500 µL of ultrapure water and 500 µL of methanol. Subsequently, ultrasound-assisted extraction (UAE) was carried out. The operative conditions were 60 °C for 20 min at full power.

### 3.2. HPLC-DAD-MS

The identification and quantification of phenolic compounds were conducted through HPLC-DAD-MS analysis. The HPLC apparatus consisted of two PU-2080 PLUS chromatographic pumps, a DG-2080-54 line degasser, a mix-2080-32 mixer, UV, diode array (DAD) detector, a mass spectrometer (MS) detector (expression compact mass spectrometer (CMS), Advion, Ithaca, NY, USA), an AS-2057 PLUS autosampler, and a CO-2060 PLUS column thermostat (all from Jasco, Tokyo, Japan). Integration was performed by ChromNAV2 chromatography software. The separation was conducted within 60 min of the chromatographic run, starting from the following separation conditions: 97% water with 0.1% formic acid, 3% methanol with 0.1% formic acid. Details of the gradient are reported in Table 2. The separation was performed on an Infinity lab Poroshell 120-SB reverse phase column (C18, 150 × 4.6 mm i.d., 2.7 µm) (Agilent, Santa Clara, CA, USA). Column temperature was set at 30 °C. The injection volume was 5 µL. Quantitative determination of phenolic compounds was performed via DAD detector at 254 nm, through 7-point calibration curves, with linearity coefficients (R2) > 0.999, in the concentration range 2–140 µg/mL. The area under the curve from the HPLC chromatogram was used to quantify the analyte concentrations in the extract. Details of the phytochemical identification are included in Table 3. The extract was also qualitatively analyzed with an MS detector in positive and negative ion mode. MS signal identification was realized through comparison with a standard solution and MS spectra present in the MassBank Europe database. The statistical analysis was performed using GraphPad Prism version 5.01 software (San Diego, CA, USA).

### 3.3. Cell Cultures

The effects of the extract (100–500 µg/mL) on myocyte C2C12, human prostate PC3 cancer, and human ovary OVCAR-3 cancer cell viability were determined through the 3-(4,5-dimethylthiazol-2-yl)-2,5-diphenyltetrazolium bromide (MTT) test. The experimental conditions are fully described in our previous papers [35,36].

### 3.4. Ex Vivo Studies

Adult C57/BL6 and female mice (3-month-old, weight 20–25 g) were housed in Plexiglass cages (2–4 animals per cage; 55 cm × 33 cm × 19 cm) and maintained under standard laboratory conditions (21 ± 2 °C; 55 ± 5% humidity) on a 14/10 h light/dark cycle, with ad libitum access to water and food. Housing conditions and experimentation procedures were strictly in agreement with the European Community ethical regulations (EU Directive no. 26/2014) on the care of animals for scientific research. In agreement with the recognized principles of “replacement, refinement and reduction in animals in research”, colon specimens were obtained as residual material from vehicle-treated mice randomized in our previous experiments, approved by the local ethical committee (‘G. d’Annunzio’ University, Chieti, Italy) and Italian Health Ministry (Project no. 885/2018-PR).

Isolated prostate and ovary specimens were maintained in a humidified incubator with 5% CO_2_ at 37 °C for 4 h (incubation period), in RPMI buffer with added bacterial LPS (10 µg/mL), as previously described [17]. During the incubation period, the tissues were challenged with scalar concentrations of the extract (10–2000 µg/mL).

### 3.5. Gene Expression Analysis

Total RNA was extracted from both prostate and ovary specimens using TRI reagent (Sigma-Aldrich, St. Louis, MO, USA), according to the manufacturer’s protocol, and reverse transcribed using a High Capacity cDNA Reverse Transcription Kit (Thermo Fischer Scientific, Waltman, MA, USA). Gene expression of TNF-α, IL-6, CAT, and SOD was determined by quantitative real-time PCR using TaqMan probe-based chemistry, as previously described [14]. PCR primers and TaqMan probes were purchased from Thermo Fisher Scientific Inc. The elaboration of data was conducted with the Sequence Detection System (SDS) software version 2.3 (Thermo Fischer Scientific). Relative quantification of gene expression was performed by the comparative 2^−∆∆Ct^ method [37].

### 3.6. In Silico Studies

Human proteins targeted by extract components were predicted using the bioinformatics platform STITCH. Docking calculations were conducted using AutoDock Vina PyRx 0.8 software. Crystal structure of the target protein was derived from the Protein Data Bank (PDB) with PDB ID as follows: 7LR0 (TRPV1). In order to prepare the protein for the docking simulation, all the water molecules and the co-crystalized heteromolecules were removed, followed by addition of hydrogen atoms and neutralization using Kollman united-atom charges. The dimensions of the grid box were 60 × 60 × 60 with 0.375 Å distance between points. Autodock4 and Lamarckian genetic algorithms were used to dock 250 conformations for each test compound (Molinspiration database). The Discovery Studio 2020 visualizer was employed to investigate the protein–ligand non-bonding interactions.

### 3.7. Statistical Analysis

Statistical analyses were performed using GraphPad Prism version 5.01 software (San Diego, CA, USA). Means ± S.E.M. were determined for each experimental group and analyzed by one-way analysis of variance (ANOVA), followed by Newman–Keuls comparison multiple test. Statistical significance was set at *p* < 0.05. The number of animals randomized for each experimental group was calculated on the basis of the “resource equation” N = (E + T)/T (10 ≤ E ≤ 20) [38].

### 3.8. Color Analysis

The samples under examination (olive oil as vehicle present at about 46% in the formulation, and PollenAid Plus Fill as formulated product used to fill the capsules) were subjected to colorimetric analysis using a spectrometer X-Rite, equipped with full-spectrum D65 illuminant and an observer angle and an observer angle of 10°. Cylindrical coordinates C*_ab_ and h_ab_ were calculated from a* and b* as is customary in the literature [15].

## 4. Conclusions

In the present study, phytochemical and pharmacological investigations were conducted on an innovative formulation of PollenAid Plus Soft Gel capsules, which contain phenolic compounds with well-established anti-inflammatory and antioxidant activities. The relative instability shown by the colorimetric analyses performed at nine months of storage on the mixture used to fill the soft capsules, probably due to a carotenoid bleaching, indicates the importance of protecting the obtained mixture. For this reason, the formulation is contained in amber soft gel capsules. This formulation also covers the unappealing dark brown color of the mixture.

For the other analyses and to perform the biological assays, a hydroalcoholic extract was prepared from this commercial formulation and tested in the experimental paradigm. In this regard, the extract was rich in phenolic compounds, with 3-hydroxytyrosol, catechin, gentisic acid, and chlorogenic acid being the prominent phytochemicals. In in vitro models constituted by prostate and ovary cancer cells, the extract altered cell viability only at the highest concentration, with a slight reduction that cannot be considered toxic because it was <30% compared to the control treated group. However, in isolated prostate and ovary specimens exposed to LPS, the extract displayed significant reduction of IL-6 and TNF-α gene expression, which demonstrate anti-inflammatory effects. In the same ex vivo models, the extract was effective in restoring the mRNA levels of both SOD and CAT, which are deeply involved in the endogenous antioxidant mechanism. Lastly, an in silico study predicted the putative targets of the main phytochemicals present in the extracts. Particularly, catechin was predicted to be the main phenolic compound influencing the anti-inflammatory effects of the extract, whereas 3-hydroxytyrosol, chlorogenic acid, and gentisic acid could be the main phytochemicals responsible for the slight reduction of cell viability induced by the extract, at the highest tested concentration. Overall, the present findings demonstrated anti-inflammatory and antioxidant effects of this formulation in both prostate and tissue; thus, suggesting its capability in the management of the inflammatory component of both bacterial prostatitis and PID. Intriguingly, docking runs suggest the TRPV1 as a putative target, and the predicted micromolar interactions between catechin and chlorogenic acid towards this receptor support future studies for a better comprehension of the molecular mechanisms.

## Figures and Tables

**Table 1:** CIEL*a*b* parameters of the carrier olive oil and of the filling formulated product.

	Olive Oil	PollenAid Plus Fill	
L*	70.46	14.96	
a*	5.55	5.42	
b*	111.60	13.87	
C*_ab_	111.74	14.89	
h_ab_	87.15	68.67	
ΔL*		Respect to olive oil:	−55.50 Darker
		Respect to Graminex pollen powder:	−71.09 Darker
Δa*		Respect to olive oil:	−0.14 More green
		Respect to Graminex pollen powder	+3.71 More red
Δb*		Respect to olive oil:	−97.73 More blue
		Respect to Graminex pollen powder:	+1.91 More yellow
ΔC*_ab_		Respect to olive oil:	−96.84 More opaque
		Respect to Graminex pollen powder:	+2.80 Less opaque
Δh_ab_		Respect to olive oil:	−8.48 More red
		Respect to Graminex pollen powder:	−13.21 More red
ΔE		Respect to olive oil:	+112.39
		Respect to Graminex pollen powder:	+71.21

**Table 2:** Gradient elution conditions.

TIME (min)	Composition A% (Water + Formic Acid 0.1%)	Composition B% (Methanol + Formic Acid 0.1%)	Flow (mL/min)
1	97	3	0.6
5	77	23	0.6
12	73	27	0.6
18	57	43	0.6
25	52	48	0.6
32	50	50	0.6
34	50	50	0.6
37	35	65	0.6
40	5	95	0.6
47	10	90	0.6
48	10	90	0.6

**Table 3:** Mass to charge (*m*/*z*) ratios, retention times, and quantities of the investigated phenolic compounds. DAD detector was set at 254 nm.

	Standard	*m*/*z*	Retention Time (min)
1	Gallic acid	170.15	8.967
2	3-Hydroxytyrosol	154.16	11.85
3	Caftaric acid	312.23	13.19
4	Catechin	290.27	15.383
5	Gentisic acid	154.12	16.147
6	4-Hydroxybenzoic acid	138.12	16.633
7	Loganic acid	376.36	17.257
8	Chlorogenic acid	354.31	17.66
9	Vanillic acid	168.15	19.22
10	Caffeic acid	180.16	19.73
11	Epicatechin	290.27	19.973
12	Syringic acid	198.17	20.673
13	Syringaldehyde	182.17	22.32
14	*p*-Coumaric acid	164.16	23.73
15	*t*-Ferulic acid	194.18	24.793
16	Benzoic acid	122.12	27.153
17	*t*-Cinnamic acid	148.15	35.817
18	Naringenin	272.25	38.87
19	2.3-Dimethylbenzoic acid	150.17	39.7
20	Hesperetin	302.28	40.773
21	Kaempferol	286.24	42.333
22	Carvacrol	150.22	44.393
23	Thymol	150.22	44.5
24	Flavone	222.24	45.077
25	3-Hydroxyflavone	238.24	45.36

## Data Availability

The datasets generated and analyzed in the current study are available from the corresponding author on reasonable request.

## Supplementary Materials

The following supporting information can be downloaded at: https://www.mdpi.com/article/10.3390/molecules27196279/s1, Table S1: Quantitative analysis of the extract.

## Sample Availability

Samples of the compounds are available from the authors.
