# Supplementary material for: Protective Effects of PollenAid Plus Soft Gel Capsules’ Hydroalcoholic Extract in Isolated Prostates and Ovaries Exposed to Lipopolysaccharide
Source: Molecules. 2022 Sep 23;27(19):6279. doi: 10.3390/molecules27196279 (PMC9570715; doi:10.3390/molecules27196279)
Supplement: Supplementary file 1 [file molecules-27-06279-s001.zip › molecules-1918786-supplementary.pdf]

Table S1. Quantitative analysis of the extract.

	Standard	*m*/*z*	Retention Time (min)	Quantity (µg/mL)	Standard error (SE)
1	Gallic acid	170.15	8.967	0.199	0.01
2	3-Hydroxytyrosol	154.16	11.85	18.040	0.49
3	Caftaric acid	312.23	13.19	1.412	0.06
4	Catechin	290.27	15.383	7.207	0.04
5	Gentisic acid	154.12	16.147	3.440	0.04
6	4-Hydroxybenzoic acid	138.12	16.633	N/A	
7	Loganic acid	376.36	17.257	0.523	0.01
8	Chlorogenic acid	354.31	17.66	2.807	0.05
9	Vanillic acid	168.15	19.22	N/A	
10	Caffeic acid	180.16	19.73	N/A	
11	Epicatechin	290.27	19.973	0.286	0.01
12	Syringic acid	198.17	20.673	0.122	0.01
13	Syringaldehyde	182.17	22.32	2.400	0.03
14	*p*-Coumaric acid	164.16	23.73	1.166	0.01
15	*t*-Ferulic acid	194.18	24.793	0.248	0.01
16	Benzoic acid	122.12	27.153	3.122	0.04
17	*t*-Cinnamic acid	148.15	35.817	N/A	
18	Naringenin	272.25	38.87	0.458	0.02
19	2.3-Dimethylbenzoic acid	150.17	39.7	N/A	
20	Hesperetin	302.28	40.773	0.925	0.05
21	Kaempferol	286.24	42.333	N/A	
22	Carvacrol	150.22	44.393	0.59	0.05
23	Thymol	150.22	44.5	0.873	0.02
24	Flavone	222.24	45.077	N/A	
25	3-Hydroxyflavone	238.24	45.36	N/A	
